# Cell death following the loss of ADAR1 mediated A-to-I RNA editing is not effected by the intrinsic apoptosis pathway

**DOI:** 10.1038/s41419-019-2160-6

**Published:** 2019-12-04

**Authors:** Carl R. Walkley, Benjamin T. Kile

**Affiliations:** 1 St. Vincent’s Institute of Medical Research, Fitzroy, VIC 3065 Australia; 2 Department of Medicine, St. Vincent’s Hospital, Melbourne Medical School, University of Melbourne, Fitzroy, VIC 3065 Australia; 3 Mary MacKillop Institute for Health Research, Australian Catholic University, Melbourne, VIC 3000 Australia; 4 Department of Anatomy and Developmental Biology, Monash Biomedicine Discovery Institute, Monash University, Melbourne, VIC 3800 Australia; 5 Present Address: University of Adelaide, Adelaide, SA 5000 Australia

**Keywords:** Cell death, Embryology

## Abstract

Modifications of RNA, collectively termed as the epitranscriptome, are widespread, evolutionarily conserved and contribute to gene regulation and protein diversity in healthy and disease states. There are >160 RNA modifications described, greatly exceeding the number of modifications to DNA. Of these, adenosine-to-inosine (A-to-I) RNA editing is one of the most common. There are tens of thousands of A-to-I editing sites in mouse, and millions in humans. Upon translation or sequencing an inosine base is decoded as guanosine, leading to A-to-G mismatches between the RNA and DNA. Inosine has different base pairing properties to adenosine and as a result editing not only alters the RNA code but can also change the RNA structure. In mammals A-to-I editing is performed by ADAR1 and ADAR2. A feature of murine loss of function ADAR1 alleles is cell death and a failure to survive embryogenesis. *Adar1*^−/−^ and editing deficient (*Adar1*^*E861A/E861A*^) mice die between E11.75–13.5 of failed hematopoiesis. Strikingly this phenotype is rescued by the deletion of the cytosolic dsRNA sensor MDA5 or its downstream adaptor MAVS, a mechanism conserved in human and mouse. Current literature indicates that the loss of ADAR1 leads to cell death via apoptosis, yet this has not been genetically established. We report that blockade of the intrinsic (mitochondrial) apoptosis pathway, through the loss of both BAK and BAX, does not rescue or modify the cellular phenotype of the fetal liver or extend the lifespan of ADAR1 editing deficient embryos. We had anticipated that the loss of BAK and BAX would rescue, or at least significantly extend, the gestational viability of *Adar1*^*E861A/E861A*^ embryos. However, the triple mutant *Adar1*^*E861A/E861A*^ *Bak*^−/−^ *Bax*^−/−^ embryos that were recovered at E13.5 were indistinguishable from the *Adar1*^*E861A/E861A*^ embryos with BAK and BAX. The results indicate that cell death processes not requiring the intrinsic apoptosis pathway are triggered by MDA5 following the loss of ADAR1.

Adenosine-to-inosine (A-to-I) RNA editing is one of the most prevalent post-transcriptional modifications of RNA. There are tens of thousands of A-to-I editing sites in mouse, and millions in humans. Upon translation or sequencing an inosine base is decoded as guanosine, leading to A-to-G mismatches between the RNA and genomic DNA. Inosine has different thermodynamic base pairing properties to adenosine and as a result A-to-I editing not only alters the RNA code but can lead to changes in RNA structure^1^. Editing frequencies vary from <1 to 100% for any given site, and dynamic differences are observed for the same site across cell types, development, and aging. A-to-I editing in an exonic region can change the protein coding potential of a transcript. However, most editing occurs in non-coding regions where it can alter splicing, miRNA binding, miRNA sequences themselves, formation of other RNA species such as circular RNAs, secondary structure, and immunogenicity. The largest proportion of mammalian editing occurs in repetitive elements such as SINEs and LINEs, including *Alu* elements, which can form structured long double-stranded RNAs (dsRNA)^1^.

In mammals, A-to-I editing is catalyzed by ADAR1 and ADAR2. ADAR1 is broadly expressed across cell types and tissues, while ADAR2 has a more restricted expression pattern with highest levels in the brain. In mice, deletion of ADAR2 causes post-natal lethality due to the development of seizures. This phenotype was rescued by homozygosity for a single residue A-to-G replacement in the genomic DNA of the A-to-I edited Q/R codon of *Gria2*, which mimics constitutive recoding at this site^2^. In contrast, *Adar1*^−/−^ or editing-deficient (*Adar1*^*E861A/E861A*^) mice die in utero at E11.75–E12.5 (refs. ^3,4^) and E13.5 (ref. ^5^), respectively. *Adar1*^−/−^ animals die from a failure of fetal liver hematopoiesis, primarily erythropoiesis, as evidenced by the fact that the phenotype is reproduced in mice with an erythroid lineage-restricted deletion of ADAR1 (ref. ^6^). ADAR1 null or editing-deficient animals can be rescued by concurrent loss of the cytosolic dsRNA sensor MDA5 (encoded by *Ifih1*) or its downstream effector MAVS^5,7,8^. This body of work, complemented and confirmed by analysis of human ADAR1-deficient cells, has established that the essential species-conserved function of ADAR1 editing is to attenuate the immunogenic potential of endogenous dsRNA and prevent an MDA5-mediated innate immune response to self-dsRNA^5,7–9^. What remains unclear are the mechanisms that cause ADAR1-editing-deficient cells to die. Strikingly, the death of ADAR1-deficient mouse embryos or ADAR1-deficient human cells can be suppressed by loss of MDA5 or MAVS, indicating that the cell death is downstream of activation of the cytosolic innate immune sensing system^5,7^.

Current theory holds that ADAR1-deficient cells die via apoptosis. The original evidence for this was obtained by histological analysis (pyknotic nuclei, DNA fragmentation) and immunohistochemical TUNEL staining of *Adar1*^−/−^ embryos at E11.0–E11.5 (refs. ^3,4^). The same gross morphological phenotype was observed in *Adar1*^*E861A/E861A*^ embryos, which specifically lack the RNA editing activity of ADAR1 (ref. ^5^), or in embryos lacking the cytosolic ADAR1p150 isoform^10^. Subsequent analysis of mice with a deletion of ADAR1 in adult hematopoietic cells or B-cells demonstrated a substantive increase in Annexin-V-positive cells in the bone marrow^11,12^. In human cells, ADAR1 knockdown or knockout has been reported to induce cell death and apoptosis by Caspase-3/7 fluorometric assays, cleaved PARP or cleaved caspase-3 western blot analysis following perturbation with either UV exposure or treatment with interferon-β^9,13^.

To definitively establish if the intrinsic (or “mitochondrial”) apoptosis pathway is required for the death of ADAR1-editing-deficient embryos, we intercrossed *Adar1*^*E861A/E861A*^ and *Bak*^−/−^*Bax*^*+/−*^ mice to generate triple mutant *Adar1*^*E861A/E861A*^ *Bak*^−/−^*Bax*^−/−^ animals. BAK and BAX are the essential effectors of the intrinsic apoptosis program. Upon activation, BAK and BAX oligomerize in the mitochondrial outer membrane, causing permeabilization and the release of apoptogenic factors^14^. We observed no *Adar1*^*E861A/E861A*^*Bak*^−/−^*Bax*^−/−^ animals at weaning from intercrosses of *Adar1*^*E861A/+*^*Bak*^−/−^*Bax*^*+/*^− mice, a not unexpected result given the perinatal lethality associated with double deficiency of BAK and BAX^15^. We therefore examined embryos at E13.5, the timepoint at which *Adar1*^*E861A/E861A*^ embryos become non-viable. From an analysis of 82 embryos across 10 litters, we recovered 2 *Adar1*^*E861A/E861A*^*Bak*^−/−^*Bax*^−/−^, compared to an expected 5 assuming Mendelian inheritance (Fig. 1a). Surprisingly, loss of BAX and BAK did not extend the survival of ADAR1-editing-deficient embryos.

**Fig. 1:**
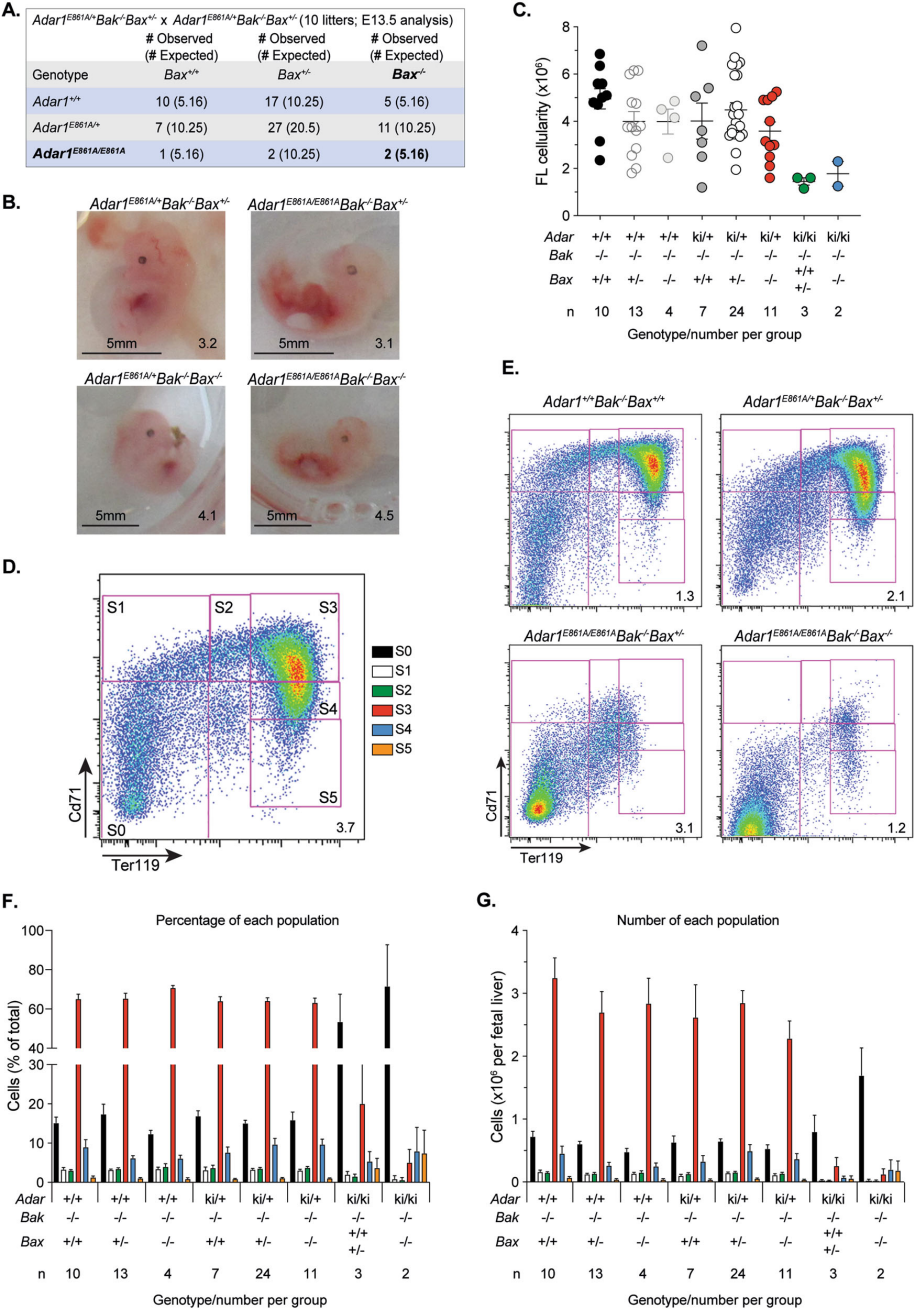
ADAR1-editing-deficient cells do not die by BAK-BAX-dependent intrinsic apoptosis. **a** *Adar1*^*E861A/+*^ animals (Adar^tm1.1Xen^, MGI:5805648) were intercrossed with *Bak*^−/−^*Bax*^*+/−*^ animals to generate *Adar1*^*E861A/+*^*Bak*^−/−^*Bax*^*+/*−^ founders (Ethics number: 2015.008; Walter and Eliza Hall Institute Animal Ethics Committee). All animals were on a C57Bl/6 background. Timed mating was undertaken, and embryos were collected at embryonic day 13.5 (E13.5). Embryos were isolated and genotyped using previously published protocols. **b** Representative photos of the embryos, genotype as indicated; embryo number indicated in the bottom right of each panel. **c** The fetal liver was isolated and single-cell suspensions prepared by passing through a 26g needle/1 mL syringe into PBS. Single-cell suspensions were counted on a Sysmex K-1000 hematological analyzer. **d** Fetal liver cells were stained with anti-mouse Cd71 (APC conjugate; Clone: R17217; eBioscience) and anti-mouse Ter119 (PE conjugated; Clone: TER-119; BD Pharmingen). Representative flow cytometry plot from an *Adar1*^*+/+*^*Bak*^−/−^*Bax*^*+/+*^ embryo with the developmental trajectory of erythroid cells in the fetal liver progressing from S0 to S5 (mature erythroid cells). **e** Representative flow cytometry plots from the indicated genotypes/embryos. **f** Quantitation of the proportion of cells in the fetal liver in each population indicated in panel **d**. Data expressed as mean ± SEM; *n* as indicated. **g** The number of cells in each population of each genotype. Data expressed as mean ± SEM; *n* as indicated.

*Adar1*^*E861A/E861A*^*Bak*^−/−^*Bax*^−/−^ embryos were small, pale, and exhibited signs of hemorrhage, similar to *Adar1*^*E861A/E861A*^*Bak*^−/−^*Bax*^*+/+*^ and *Adar1*^*E861A/E861A*^*Bak*^−/−^*Bax*^*+/−*^ embryos (Fig. 1b). Given the well-described phenotype of ADAR1-editing-deficient hematopoietic cells, and fetal livers in particular, we focused analysis on this organ. We found there was no modification of the *Adar1*^*E861A/E861A*^ phenotype by concurrent deletion of BAK and BAX. The total cellularity (Fig. 1c) and differentiation trajectory of erythroid cells (Fig. 1d–g), the primary cell type in the fetal liver at this developmental timepoint, were not changed. There was a profound loss of cells—proportionally and in absolute number—from all stages of erythroid differentiation in the ADAR1-editing-deficient mutants, irrespective of the capacity to effect intrinsic apoptosis (Fig. 1f, g).

The genetic analysis demonstrates that the intrinsic apoptosis pathway is not responsible for the death of ADAR1-editing-deficient embryos. We had anticipated that the loss of BAK and BAX would rescue, or at least significantly extend, the gestational viability of ADAR1^E861A/E861A^ embryos. However, the triple mutant *Adar1*^*E861A/E861A*^*Bak*^−/−^*Bax*^−/−^ embryos that were recovered at E13.5 were indistinguishable from embryos with wild-type BAK and BAX. In vivo the complete loss of ADAR1 or the specific abolition of RNA editing by ADAR1 are incompatible with embryonic development, yet the present results demonstrate that this not the result of utilization of the intrinsic apoptosis pathway. The MDA5–MAVS axis is the primary in vivo sensor of endogenous unedited RNA in both human and mouse cells, activating a cascade that ultimately results in cell death^5,7,8^. Several recent studies, principally from in vitro tissue culture, have begun to illuminate pathways downstream of MAVS that may be important^16–18^. Most directly addressing this, it was reported that RNaseL was the downstream effector of cell death of cells engineered to be *ADAR*^−/−^ based on rescue of the viability using human A549 cells; however, the mechanism of cell death was not clearly ascribed^18^. The ability of RNaseL knockout to rescue the murine ADAR1 deficiency has not been formally reported to the best of our knowledge. Indirectly, using transfection of the exogenous RNA agonists 5′triphosphate RNA (RIG-I agonist) or poly(I):poly(C) (TLR-3/MDA5 ligand), which signal via MAVS, it was demonstrated that in murine macrophages these stimuli induced TNF and IFNAR dependent necroptosis^16^. In vivo, the loss of IFNAR (*Ifnar*^−/−^) or the compound loss of IFNAR and IFNGR (*Ifnar*^−/−^*Ifngr*^−/−^) delayed to E15.5 the lethality of the *Adar1*^−/−^ embryos^6,8^. This suggested a role for IFN signaling in accelerating the death of ADAR1-deficient embryos, consistent with the in vitro studies^16^. The combined loss of TNF and IFNAR signaling in vivo has not been reported to date. A separate study has proposed that transfection with poly(I):poly(C) induced autophagy downstream of MAVS^17^. Recent evidence of cleaved capsase-3 or PARP in human ADAR1 null or knockdown cells has come in the context of additional exogenous stimulation, such as UV exposure or treatment with interferon-β^9,13^. Our studies reflect the cellular response to a lack of ADAR1 mediated A-to-I editing in the context of normal physiological development in vivo. Collectively, the results from this study and those reported in the literature indicate that cell death following the loss of ADAR1 is likely the result of the activation of multiple pathways, including but not solely restricted to the characteristic Type I interferon response. These include necroptosis and forms of cell death associated with inflammation^19^. The results indicate that cell death processes not requiring the intrinsic apoptosis pathway are triggered by MDA5 following the loss of A-to-I editing.
